# Clinical impact of early post-transplant red cell transfusions in kidney transplantation: a systematic review and meta-analysis

**DOI:** 10.3389/frtra.2023.1215130

**Published:** 2023-07-10

**Authors:** Sevda Hassan, Sarah Gleeson, Tina Thomson, Katrina J. Spensley, Frank Dor, Colin Brown, Fiona Regan, Liset H. M. Pengel, Michelle Willicombe, David J. Roberts

**Affiliations:** ^1^ Imperial College Renal and Transplant Centre, Imperial College Healthcare NHS Trust, Hammersmith Hospital, London, United Kingdom; ^2^ Centre for Inflammatory Disease, Department of Immunology and Inflammation, Imperial College London, Hammersmith Campus, London, United Kingdom; ^3^ Histocompatibility and Immunogenetics, NHS Blood and Transplant, London, United Kingdom; ^4^ Blood Transfusion, NHS Blood and Transplant, London, United Kingdom; ^5^ Peter Morris Centre for Evidence in Transplantation, Nuffield Department of Surgical Sciences, University of Oxford, Oxford, United Kingdom; ^6^ BRC Haematology Theme, Radcliffe Department of Medicine, and Department of Haematology Oxford, John Radcliffe Hospital, Oxford, United Kingdom; ^7^ NHS Blood and Transplant, John Radcliffe Hospital, Oxford, United Kingdom

**Keywords:** blood transfusion, transplant, kidney, outcomes, post-transplant

## Abstract

**Introduction:**

Red blood cell transfusions (RBCT) represent a potentially modifiable risk factor for HLA sensitisation and adverse outcomes post transplantation. Evidence of the clinical impact of post-transplant RBCT has been infrequently reported. Herein, we performed a systematic review of available literature to assess the prevalence of RBCT post kidney transplant, and the effect of transfusion on transplant outcomes.

**Methods:**

We included studies from 2000 to July 2022, published on Medline, Embase and the Transplant Library.

**Results:**

Ten studies were analysed which included a total of 32,817 kidney transplant recipients, with a median transfusion prevalence of 40% (range 18-64%). There was significant heterogeneity between studies in terms of patient and allograft characteristics, immunological risk, and immunosuppression protocols. Analysis of unadjusted outcomes showed that post-transplant RBCTs are associated with inferior patient survival, allograft loss, rejection and donor specific antibodies. Adjusted outcomes were described where available, and supported the adverse associations seen in the unadjusted models in many studies.

**Discussion:**

This review demonstrates that RBCT post-transplant are common and maybe associated with inferior outcomes, highlighting the urgent need for high quality prospective evidence of the effect of RBCTs on transplant outcomes.

**Systematic Review Registration:**

https://www.crd.york.ac.uk/prospero/, identifier, CRD42022348763767.

## Introduction

With only marginal improvements in long-term kidney allograft survival over time, the identification and intervention of modifiable risk factors contributing to unfavourable transplant outcomes would be welcomed (1). Whilst significant developments in research innovation have translated into clinical practice, e.g., organ pre-conditioning prior to engraftment; for other unmet needs, such as the successful treatment of chronic antibody mediated rejection, answers remain elusive (2).

Despite recognition that red blood cell transfusions (RBCTs) can provoke alloimmune anti-HLA antibodies and should be avoided in potential transplant recipients on waiting lists, less focus has been given to their potential impact on stimulation of these alloimmune responses and outcomes regarding graft and patient survival post-transplant (3). By HLA typing blood donors of RBCTs given to post-transplant recipients and defining the presence and allotype of the respective anti-HLA response, our group has previously shown that *de novo* HLA sensitisation to foreign HLA antigens from allogenic blood donors may occur and can contribute to alloimmune injury and transplant loss (4). Since the publication of this report, several further observational studies have been published assessing the significance of post-transplant RBCTs (5–11).

We perform here a systematic review and meta-analysis of the available evidence of the clinical impact of allogenic RBCT given in the early post-transplant period. This analysis had the following aims: first, to assess the reported prevalence of post-transplant RBCTs; second, to analyse all available evidence of the effect of RBCT on transplant outcome; and finally, to critically assess the quality of this evidence, appraising evidence gaps if present.

## Materials and methods

### Inclusion criteria

Eligible studies included those that compared RBC transfusion with no RBC transfusion, where transfusion occurred either intra-operatively, perioperatively, or post-operatively up to one year post transplant. We excluded all studies where donor-specific or pre-transplant transfusions were given as part of the identified transplant episode. We only included cohorts with adult kidney transplant recipients (both living and deceased donor transplants), permitting those that included recipients of simultaneous kidney-pancreas transplants, but excluding studies assessing RBCTs in other solid organ transplant groups. Cohorts reporting antibody incompatible transplants alone were excluded, but were included where antibody incompatible transplants represented a minority of the population or included HLA incompatibility defined by the presence of low level pre-transplant DSA, in the absence of desensitisation. Given the developments in the field of HLA antibody detection and histopathological diagnoses over the past two decades, we only included studies that reported patient cohorts transplanted after the year 2000. Only studies published in the English language were considered. All study types were included if they otherwise met the inclusion criteria.

The outcomes of interest were allograft survival, patient survival, rejection (type not specified), and *de novo* donor specific antibodies (DSA). The study was designed and reported as per PRISMA guidelines (12).

### Search strategy

The electronic databases Medline (Ovid), Embase (Ovid), and the Transplant Library (Ovid) were searched for publications between 2000 and the 20th of July 2022, using a combination of subject headings and keywords for kidney transplantation, blood transfusion, and survival outcomes (LP).

### Review methods and data extraction

Initial screening included review of titles and abstracts to select only those studies that satisfied the inclusion criteria, which was performed by two independent reviewers (SG, TT). This was followed by a full-text review of all possible eligible studies, which was subsequently refined, leaving the final selected studies for inclusion (SG, TT, MW). Any discrepancies which arose were resolved following a discussion and agreed consensus.

Data were extracted from the final selected studies by two independent reviewers (MW, KS). Where there were multiple studies reporting the same dataset, only one (with the most complete dataset) was included. Data were extracted on the outcomes of interest in a predefined proforma. Additional data were also collected via free text, which, in the opinion of the reviewers, would be relevant to report. The Risk Of Bias In Non-randomized Studies of Exposure (ROBINS-E) tool was used to assess the risk of bias of included studies (MW, KS) (13).

### Statistical analysis

Meta-analyses were performed using the statistical software Review Manager 5.4.1. Each outcome measure was summarised using Odd Ratios (OR) with 95% confidence intervals (CI) as reported, and irrespective of length of study follow up. All outcomes required a minimum number of four studies to be analysed. We assessed both unadjusted and adjusted outcome data. Where data were unavailable or present in a format that rendered it unextractable for analysis, a descriptive summary of results was performed. Statistical heterogeneity was assessed using *I*^2^ (a value >50% was considered to represent significant heterogeneity); where there was heterogeneity, we used the Mantel–Haenszel random-effects methods to assess outcomes.

The protocol was registered with PROSPERO (CRD42022348767).

## Results

**Figure 1:**
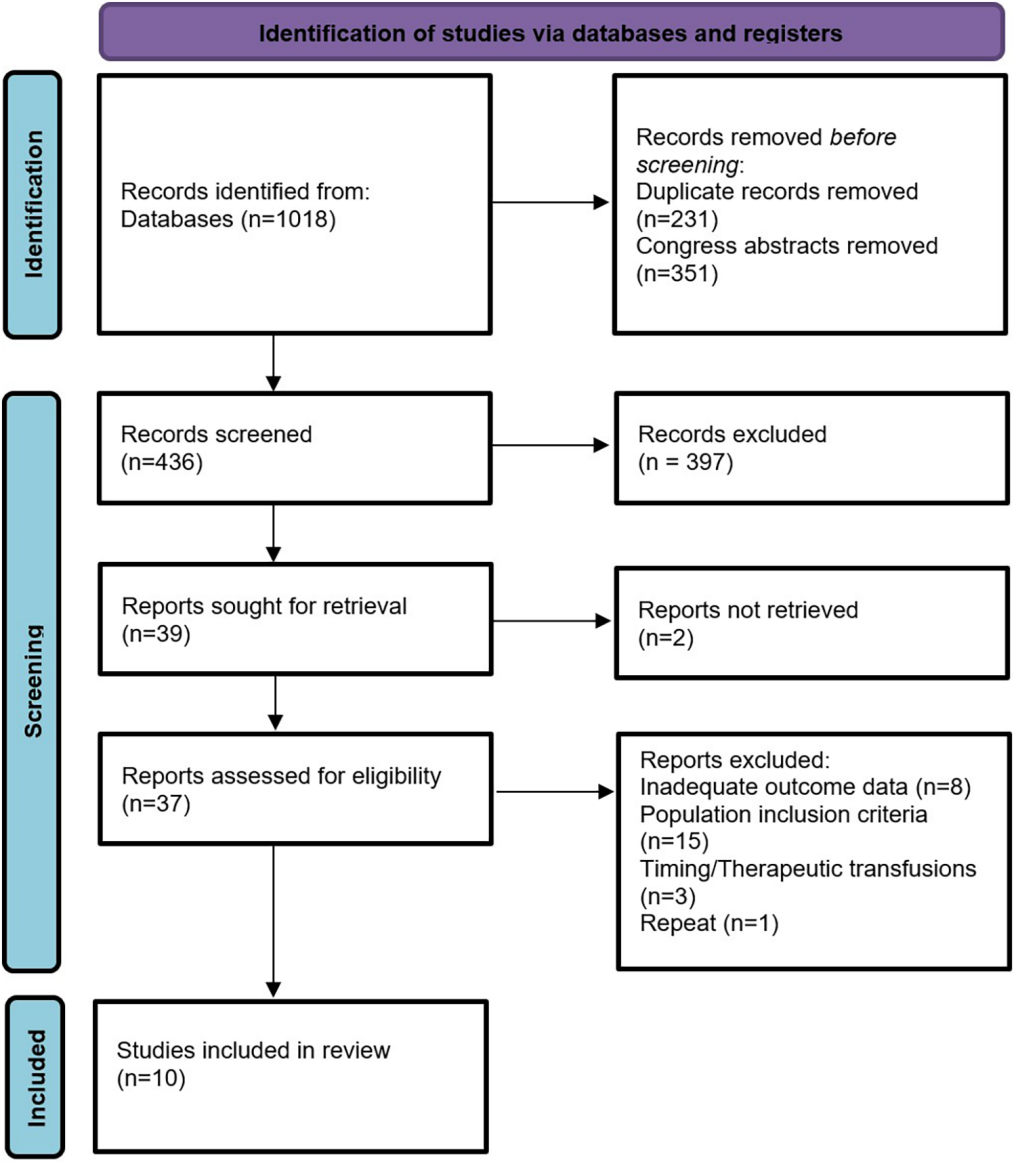
PRISMA flow diagram of systematic review.

From 1,018 records identified in the database search, 436 were screened for eligibility, see Figure 1. Full-text assessment was undertaken in 37 studies, of which 10 remained eligible for inclusion for data extraction. Details of the 27 studies excluded from the analysis may be found in Supplementary Material Table S1.

### Study characteristics

A summary of the study characteristics is shown in Table 1 (4–11, 14, 15). All studies were observational cohort studies; three were multicentre and seven were single centre studies. The total number of patients included across the studies was 32,817 (range 258–13,871). The median prevalence of transfusion was 40% (range 18%–64%). The reported period for transfusion varied considerably between the studies, with six reporting transfusions within the first 30 days only, one reporting up to 3 months, and three others reporting up to one year. These latter studies, which included transfusions up to one year, reported that most transfusions occurred within the first week post-transplant, with a median time to transfusion of 3–5.7 days post-transplant (4, 7, 14). One study only captured transfusions occurring from day one post-transplant, thereby excluding those individuals who would have received intra- or peri-operative blood (7). Eight studies reported that blood products used were leucodepleted, with absent reporting for this characteristic in the remaining two studies (5, 8).

**Table 1:** Characteristics of eligible studies.

Study	Journal	Country	Study type	Study period	Study number	Transfusion prevalence	Transfusion period	Leuco-depleted	Follow up period
Daloul et al. (6)	Kidney International Reports	US	Single Centre	2015−2017	273	127 (47%)	1st 30 days	Yes	1 year
Ferrandiz et al. (14)	American Journal of Transplantation	France	Single Centre	2008−2012	390	250 (64.1%)	1st year (Median 3 days)	Yes	1 year
Fidler et al. (15)	Transplant Immunology	Australia	Multicentre	2003–2007	258	111 (43.0%)	1st 30 days	Yes	Up to 100 months
Gaiffe et al. (11)	Frontiers in Immunology	France	Multicentre	2002–2008	12,559	3483 (28%)	1st 14 days	Yes	Up to 10 years
Hassan et al. (4)	American Journal of Transplantation	UK	Single Centre	2006–2015	1,104	677 (61.3%)	1st year (Majority 1st week)	Yes	Up to 10 years
Jalalonmuhali et al. (5)	Human Immunology	Australia	Single Centre	2010–2018	699	203 (29%)	1st week	NR	Up to 3,000 days
Jouve et al. (10)	Frontiers in Immunology	France	Single Centre	2004–2015	981	292 (29.7%)	1st 3 months	Yes	1 year
Khedjat et al. (9)	Transplant International	France	Single Centre	2007–2018	1,424	258 (18%)	1st 30 days	Yes	Median 4.52 years
Lee et al. (8)	Journal Clinical Medicine	Korea	Multicentre	2007–2016	13,871	7277 (52.46%)	Transplant Admission	NR	Up to 7 years
Massicotte-Azarniouch et al. (7)	Kidney International Reports	Canada	Single Centre	2002–2018	1,258	468 (37.2%)	From post-transplant day 1 (Median time 5.7 days)	Yes	Median 1,405 days

Where reported, the proportion of the study cohorts receiving transfusions differed according to age, gender, donor type, and induction agent used. Nine studies reported transfusion prevalence by gender, donor type, and age; eight of these studies reported that transfusions were more common in females compared with males (5–11, 14, 15). Of nine studies reporting the impact of donor type, eight found that the prevalence of transfusions was higher in recipients of deceased donor kidney transplants. Older age was associated with transfusions in eight of the nine reporting studies (5–11, 14, 15). Where data were available, the use of depleting immunosuppressive therapies was also associated with a higher prevalence of transfusions in six of seven studies (5–10, 14). Other clinical characteristics reported to be associated with a higher likelihood of transfusion in at least one study included pre-transplant HLA sensitisation (9–11, 15), time on dialysis pre-transplant (9–11), and longer cold ischaemic times (9–11, 15). These characteristics are important to consider as they may influence outcomes post-transfusion if not included in an adjusted analysis, and contributed to the risk of bias assessment, specifically the risk of confounding, participant selection, and post-exposure interventions, see Supplementary Material Table S2. A summary of the clinical characteristics included in the individual studies is shown in Table 2.

**Table 2:** Immunological characteristics of patient populations in eligible studies.

Study	Overall age	Transplant type	Donor type	Immunological risk	Induction immunotherapy	Maintenance immunotherapy	Specific exclusion	RBCT Risk independently assessed	Protocol DSA/biopsies
Daloul et al. (6)	55 (43–62)	Kidney Alone (1st grafts)	DD^a^/LD	ABOc HLAc (Non-sensitised patients only)	T-cell depleting^a^/Anti-IL2R	CNI plus mTOR^a^/CNI plus MPA Steroid sparing	Graft loss (<1 month)	Yes	No/No
Ferrandiz et al. (14)	53 ± 13	Kidney Alone	DD^a^/LD	ABOc HLAc (Non-sensitised patients only)	Anti-IL2R^a^/T-cell depleting	CNI/MPA/Steroids^a^	Early graft loss (<1 month) Graft nephrectomy (1st year)	Yes	Yes/No
Fidler et al. (15)	NR	Kidney Alone^a^/SPK	DD^a^/LD	ABOc—NR HLAi (DSA only)^d^	Anti-IL2R^a^/T-cell depleting	CNI/MPA/Steroids^a^	–	Yes	No/No
Gaiffe et al. (11)	49.1 ± 13.3	Kidney Alone (1st grafts)	DD^a^/LD	NR^	NR	NR	Graft failure/death (<14 days)	Yes (propensity match score)	NA/NA
Hassan et al. (4)	NR	Kidney Alone^a^/SPK	NR	ABOc HLAc only	Alemtuzumab^a^/Anti-IL2R	CNI monotherapy^a^/CNIplus MPA (steroid sparing)	Graft loss (<1 week)	Yes	Yes/No
Jalalonmuhali et al. (5)	83.7% < 65 years	Kidney Alone	DD^a^/LD	ABOi HLAi (DSA only)^c^ included	Anti-IL2R^a^/T-cell depleting	CNI/MPA/Steroids^a^	Graft loss (<1 month)	Yes	Yes/Yes
Jouve et al. (10)	51.76 ± 14.42	Kidney Alone	DD^a^/LD	ABOc/HLAc	T-cell depleting^a^/Anti-IL2R	CNI/MPA/Steroids^a^	Graft loss (<3 months)	Yes	Yes/Yes
Khedjat et al. (9)	NR	Kidney Alone	DD^a^/LD	HLA/ABOc only	T-cell depleting^a^/Anti-IL2R	CNI/MPA/Steroids^a^ (steroid sparing for non-sensitised)	Graft failure/death (<1 month)	Yes	Yes/NR
Lee et al. (8)	54.2% ≤ 49 years	Kidney Alone	DD/LD^a^	ABOc—NR HLAi (DSA only)^b^	Anti-IL2R^a^/T-cell depleting	NR	–	Yes	No/No
Massicotte-Azarniouch et al. (7)	52 ± 14	Kidney Alone	DD^a^/LD	ABOc/HLAc	T-cell depleting^a^ (43.1%)	CNI based (82.9%)	Transfusions from Day 1	Yes (propensity match score)	No/No

NR^, not reported if antibody incompatible included; c, compatible; i, incompatible

^a^ Dominant category.

^b^ Preformed DSA.

^c^ Preformed DSA MFI > 2,000 considered HLAi, ABOi 1.9%.

^d^ Preformed DSA, no augmented immunosuppression reported.

### Transfusion effects on allograft and patient survival

**Figure 2:**
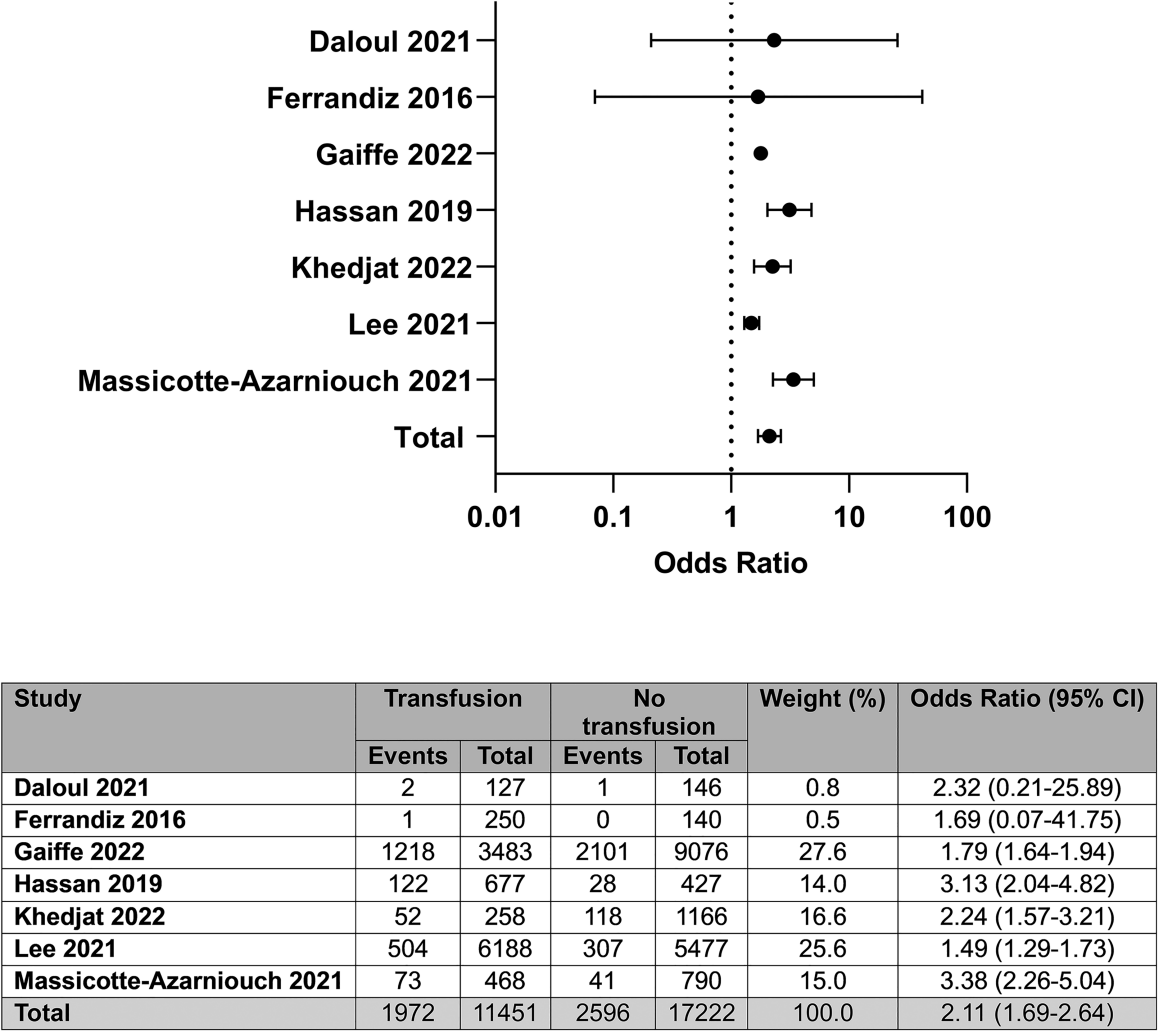
Unadjusted odds of allograft loss by blood transfusion status.

Of the ten included studies, unadjusted data on allograft survival were available in seven, representing 28,673 patients (4, 6–9, 11, 14). From these seven studies, we found that patients who receive a RBCT post-transplant have a higher odds of allograft failure compared with patients who are not transfused; OR: 2.11 (95% CI: 1.69–2.64); *I*^2^ = 74%, see Figure 2.

Only two of seven studies reporting on allograft survival found no association between transfusion and allograft loss (6, 14). Of note, although both studies included predominantly deceased donor transplant recipients, they only assessed recipients receiving antibody-compatible transplants, who were non-sensitised. Further, both studies excluded patients who lost their grafts within the first month post-transplant, with the study by Ferrandiz and colleagues also subsequently excluding patients who had to have a graft nephrectomy in the first year (9, 14). Neither study represented unselected patient cohorts, therefore introducing a potential bias in favour of superior outcomes in transfused patients.

Seven studies reported adjusted data on the association between blood transfusion and allograft loss. Three reported outcomes by post-transplant RBCT versus not, as a binary value (4, 5, 9), whilst the remaining four studies reported association by number of transfusions received or historical receipt of a blood transfusion (7, 8, 11, 15). Extractable data from the studies were not available to perform a meta-analysis; however, descriptively, five of seven studies reported post-transplant RBCTs to be associated with inferior allograft survival in adjusted models, with greater impact in those receiving an increased number of red cell units or those who had previously received a RBCT pre-transplant. The two studies reporting no association in adjusted models included binary data of the receipt of a post-transplant RBCT only (5, 9).

**Figure 3:**
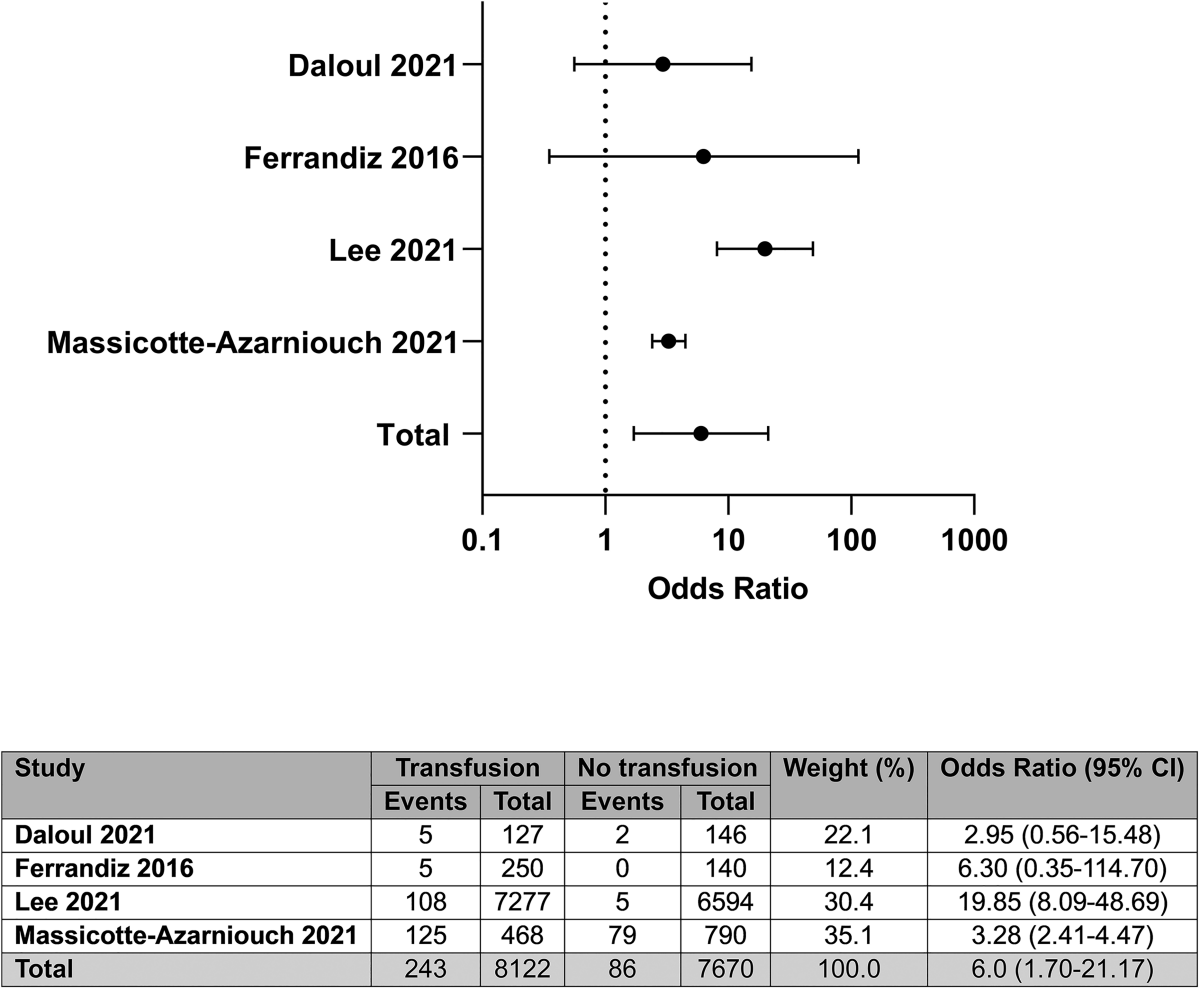
Unadjusted odds of death by blood transfusion status.

Four of the ten studies provided unadjusted data on patient survival post-transplant, representing 15,792 participants (6–8, 14). Compared with patients not needing or receiving a transfusion, a post-transplant RBCT was associated with greater odds of death; OR: 6.00 (95% CI: 1.70–21.17); *I*^2^ = 82%, see Figure 3.

The two studies that reported no impact on patient survival were the two studies that had reported no association between transfusion and allograft survival, and so have similar considerations regarding bias in favour of superior outcomes in transfused patients (6, 14). The two remaining studies that showed an association between post-transplant RBCT and death reported on risk within unselected patient cohorts (7, 8).

Four of ten studies provided adjusted data in patient survival post-transplant (6–8, 11). One reported there to be no association between post-transplant RBCT and death, where RBCT was considered as a binary value of present or absent in the adjusted model (6). The other three all reported an increased risk of death in patients receiving a post-transplant RBCT, when considering blood transfusion volume or historic blood transfusions (7, 8, 11).

### Transfusion effects on rejection and DSA

**Figure 4:**
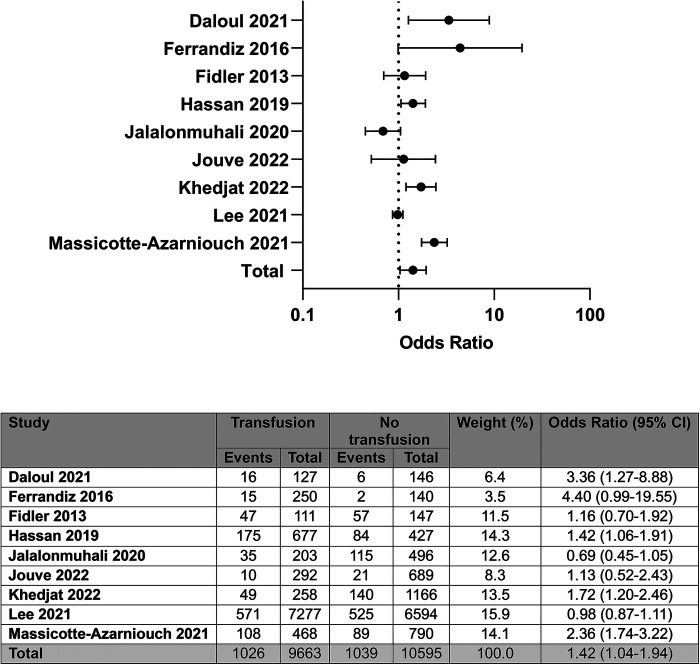
Unadjusted odds of rejection by blood transfusion status.

Nine of the ten studies contained analysable unadjusted data on rejection episodes post-transplant, representing 20,258 patients (4–10, 14, 15). Post-transplant RBCT was associated with increased odds of rejection compared with no transfusion; OR: 1.42 (95% CI: 1.04–1.94); *I*^2^ = 83%, see Figure 4.

Of these nine studies, five reported no association between transfusion and any rejection episode. Three of these five studies specifically assessed the effect of RBCT on antibody-mediated rejection (ABMR) (5, 14, 15). On so doing, two reported a significant association with ABMR, with one reporting an impact only in patients who had been re-exposed to blood post-transplant, having been transfused at some time prior to transplant (14, 15). Two of the four studies reporting an association between a RBCT and any rejection episode further differentiated between ABMR and T-cell-mediated rejection (TCMR), and both reported a positive association between post-transplant RBCT and ABMR (4, 7). It is also notable that immunosuppression data were recorded in three of the positively associated studies, and all three incorporated the use of steroid sparing protocols (4, 6, 7).

Four studies provided adjusted data on rejection episodes in recipients of a post-transplant RBCT (5, 7, 9, 14). None of the studies reported RBCTs being associated with rejection when all rejection episodes were considered; however, one reported an increased risk of ABMR in patients receiving an increasing number of RBCTs (7).

**Figure 5:**
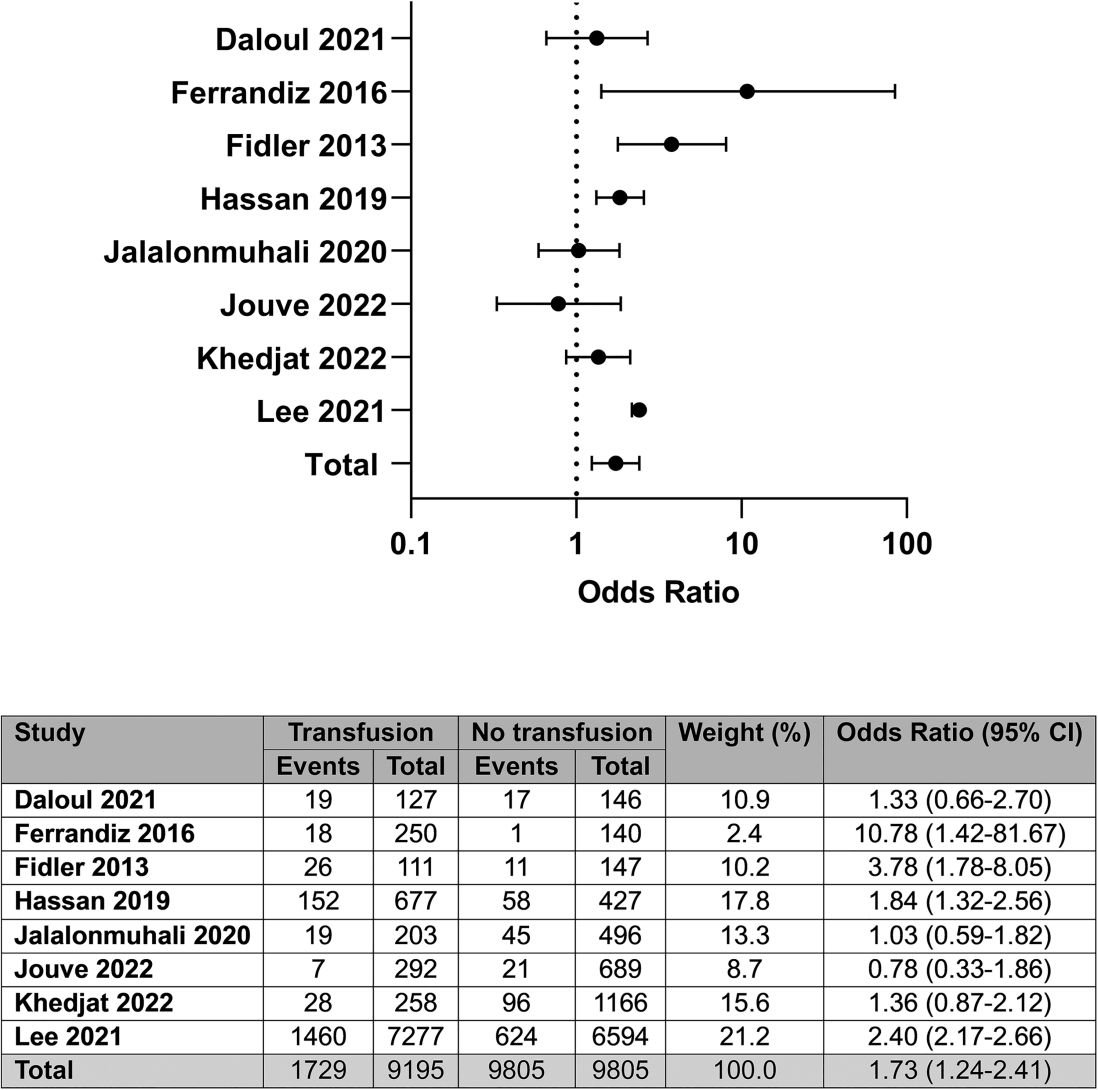
Unadjusted odds of donor specific antibody detection by blood transfusion status.

Eight of the ten studies provided unadjusted data on the detection of DSA post-transplant, representing 19,000 patients (4–6, 8–11, 14). Compared with no transfusion, post-transplant RBC transfusion was associated with a greater odds of DSA detection; OR: 1.73 (95% CI: 1.24–2.41); *I*^2^ = 74%, see Figure 5.

Of the eight studies assessing the correlation between transfusions and DSA, four studies showed a positive association between transfusion and DSA. Routine monitoring for DSA was reported to occur in two of the four studies showing an association between transfusion and DSA (4, 14), with three of the four studies failing to show a significant association (5, 9, 10). Overall, of the four studies showing a positive association between RBCT and DSA, two included recipients of HLA-incompatible transplants (8, 15), compared with one of the studies reporting no association (5). Steroid sparing protocols were included in three studies in total; one in the positively associated group (4) and two in the non-associated group (6, 9).

Five studies (4, 5, 9, 10, 14) provided adjusted data on the association between post-transplant RBCT and DSA, with two studies reporting a positive association (4, 14).

## Discussion

In this systematic review and meta-analysis of the clinical impact of post-transplant RBCT in kidney transplant recipients, we have shown that early post-transplant RBCTs are associated with adverse transplant outcomes. However, any conclusions need to be interpreted with caution given the significant heterogeneity of the studies included, both from a methodological perspective, the patient populations observed, and the limitations of the meta-analysis on adjusted data. Nevertheless, studies are consistent in the high prevalence of RBCT, which, given the lack of wider reports in the literature, suggests that the clinical significance of post-transplant RBCTs may have been unrecognised for too long. Collectively, the data within this review support the need for further evidence on why these patients are being transfused, but also to help unpick the mechanisms behind the association with poor outcomes, separating correlation from causation.

A significant limitation in the interpretation of the clinical outcomes associated with post-transplant RBCT is the considerable effect of confounding. It is easy to imagine how indications for RBCT themselves impact on outcomes, e.g., the need for a RBCT to enable a safe biopsy used to diagnose rejection, a recipient with higher haemoglobin thresholds due to significant co-morbidities having a greater risk of death, or major bleeding requiring large volume transfusions associated with technical causes of allograft loss. Attempts to circumvent these confounders in this review included strict inclusion and exclusion criteria. Many of the included studies used multivariate models to adjust for risk factors usually associated with inferior allograft outcome, therefore supporting RBCT as an independent risk factor for adverse outcomes. Unfortunately, there was inadequate extractable data from the adjusted models to perform meta-analyses of these studies. However, the significant unadjusted data, together with descriptive support from the adjusted models, support the adverse impact of post-transplant RBC, but now requires high quality evidence to confirm this one way or the other. As part of delivering this evidence, it will also be important to differentiate correlation and causation, and to understand the mechanistic pathways involved in injury, which cannot be determined from the available evidence. It is of interest that a subsequent report on the cohort analysed by Massicotte-Azarniouch and colleagues demonstrated an association between RBCT and infection risk post-transplant (16). The association is also reported in the non-transplant literature, with a meta-analyses showing restrictive transfusion practices being associated with less severe infection in hospitalised patients, though the mechanisms involved are not fully understood (17). This is an important observation given that infection is one of the leading causes of death in transplant recipients.

The evidence of RBCT as a cause of allosensitisation is strong and recognised since the early days of histocompatibility (18, 19). In the pre-transplant setting, sensitisation risk post-transfusion is believed to correlate with pre-sensitisation status and volume (or exposure) of RBCs (18, 20, 21). Despite the finding that 40% of waitlist patients are pre-sensitised prior to transplant, it has been perceived that a post-transplant RBCT has inconsequential impact on *de novo* allosensitisation and alloimmune injury (3, 19). However, direct evidence of a cause of post-transplant sensitisation by HLA typing RBC donors, together with the association shown in this review, should encourage reappraisal of the status quo (4). This is especially important given that alloimmunity remains the leading cause of transplant failure, and with no known effective treatments for chronic antibody injury, prevention remains our premier defence (22). Given the limited longevity of transplants and the growing proportion of patients awaiting repeat transplants on waiting lists, avoidance of additional sensitisation via RBCT will also be important (3). However, it should also be noted that the reported odds for DSA detection following RBCT post-transplant in this study again require caution, given the disparate patient populations and protocols. In favour of greater odds of DSA (and rejection) are those populations utilising steroid sparing immunosuppression protocols (4, 6, 9, 23, 24). A higher prevalence of DSA may also be seen in studies that undertook routine monitoring for DSA, as opposed to indicative testing, which also extends to biopsy-proven rejection episodes (4, 5, 9, 10, 14). Conversely, studies that investigated the impact of RBCT on non-sensitised transplant recipients receiving first grafts are likely to report more favourable outcomes, but it may be argued, do not broadly represent clinical practice (6, 14).

We excluded cohorts that included antibody incompatible transplants only and cohorts that investigated paediatric recipients, whether as full cohorts or contributing to the overall dataset (25–27). Therefore, further research will be required to assess the impact of RBCT in these specific subpopulations and other organ transplant groups. It is likely that such review will share the same challenges as this present one, predominantly related to the interpretation of heterogenous study populations and the methodology of reporting. Accepting this as a significant limitation of our analysis, we have provided detailed summaries of the studies included to demonstrate key differences in the reports included. We defined our criteria to keep the reporting of outcomes as consistent as possible, and only included studies reporting populations transplanted after the year 2000. This period is when post-transplant DSA monitoring started to become integrated into clinical practice; the 2001 Banff classification of allograft pathology defined antibody mediated rejection for the first time (24).

Although not a pre-defined aim of our review, we were able to summarise some identified risk factors for transfusion. This may help to identify indications for post-transplant RBCT. For example, immunosuppression with lymphocyte-depleting agents was associated with RBCT in several studies, which is biologically plausible, more likely to cause myelosuppression compared with non-depleting agents. Recipients of deceased donor kidneys were more likely to need transfusion; this makes sense again as they have greater risks of complications, including delayed graft function. Age and female gender were also reported as risk factors for RBCT on univariate analysis, which may reflect differences in red cell reserves or technical challenges.

To conclude, we have shown that early post-transplant RBCTs are common and may be associated with greater odds of adverse transplant outcomes. Despite challenges related to heterogeneity and confounding between the studies included in this systematic review and meta-analysis, we believe that the data are important and warrant further consideration; firstly, to ensure prevention of RBCT where possible, aiming to reduce the frequency of transfusion; secondly, to understand the absolute risk of transfusion on *de novo* sensitisation and to assess mechanisms to reduce this, for example, the provision of HLA matched red cells. Finally, the data help in understanding the mechanisms associated with a reduction in short-term transplant loss and death, and aid a strategy to prevent these adverse outcomes.

## Data Availability

The original contributions presented in the study are included in the article/**Supplementary Material**, further inquiries can be directed to the corresponding author.
