# Supplementary material for: Clinical impact of early post-transplant red cell transfusions in kidney transplantation: a systematic review and meta-analysis
Source: Front Transplant. 2023 Jul 10;2:1215130. doi: 10.3389/frtra.2023.1215130 (PMC11235259; doi:10.3389/frtra.2023.1215130)

**Table S1**. **Publications excluded from the analysis**

| Author | Year | Journal | Title | Exclusion Group | Reason for Exclusion | Summary of post-transplant transfusion impact |
| --- | --- | --- | --- | --- | --- | --- |
| Akcay | 2004 | Transplantation Proceedings | Angiotensin-converting enzyme genotype is a predictive factor in the peak panel-reactive antibody response | Inadequate outcomes | No post-transplant transfusions reported | NA |
| Bynum | 2018 | Transfusion | Transfusion of leukoreduced blood products and risk of antibody-mediated rejection of renal allografts | Population | HLAi patients only | No association with rejection |
| Campos | 2012 | Transplantation Proceedings | Do intraoperative hemodynamic factors of the recipient influence renal graft function? | Population | 1980-2009 | Transfusion associated with rejection and worse graft function |
| Carvalho | 2021 | Transplantation | Is There Sufficient Evidence Justifying Limited Access of Jehovah's Witness Patients to Kidney Transplantation? | Population | 1989-2018, inadequate outcomes | NA |
| Carvalho | 2018 | Acta Urologica Portuguesa | Multiple Renal Arteries in Kidney Transplantation: Is it a Problem Nowadays? | Population | 1980-2017, inadequate outcomes | NA |
| Choi | 2017 | Medicine | Risk factors and outcomes associated with a higher use of inotropes in kidney transplant recipients | Inadequate outcomes | No report of outcomes related to transfusion | NA |
| El-Agroud | 2004 | American Journal of Nephrology | Pretransplant mixed lymphocyte culture still has an impact on graft survival | Population | 1976-2002, included therapeutic transfusions | NA |
| El-Husseini | 2005 | Pediatric Transplantation | Determinants of graft survival in pediatric and adolescent live donor kidney transplant recipients: a single center experience | Population | Paediatric, 1976-2004; inadequate outcomes | NA |
| Garcia-Sanchez | 2012 | Transplantation Proceedings | Pairs of kidneys transplanted from the same donor: is there any difference? | Inadequate outcomes | No report of outcomes related to transfusion | NA |
| Gierczak | 2021 | Transplantation Proceedings | Impact of Immunosuppressive Strategies on Post-Kidney Transplantation Thrombocytopenia | Inadequate outcomes | No report of outcomes related to transfusion | NA |
| Gupta | 2019 | Experimental & Clinical Transplantation | Renal Transplant and Its Outcomes: Single-Center Experience From India | Inadequate outcomes | No post-transplant transfusions reported | NA |
| Hardy | 2001 | Clinical Transplants | Sensitization 2001 | Timing | 1992-2000, included therapeutic transfusions | NA |
| Hiesse | 2001 | Kidney International | Multicenter trial of one HLA-DR-matched or mismatched blood transfusion prior to cadaveric renal transplantation | Timing | Therapeutic transfusions pre-transplant | NA |
| Higgins | 2004 | Transplantation | Acute rejection after renal transplantation is reduced by approximately 50% by prior therapeutic blood transfusions, even in tacrolimus-treated patients | Timing | Therapeutic transfusions pre-transplant | NA |
| Kim | 2021 | Blood Transfusion | Perioperative blood usage and therapeutic plasma exchange in kidney transplantation during a 16-year period in South Korea | Repeat Data | Used same registry as Lee, K | NA |
| Lim | 2018 | BMC Nephrology | Factors associated with anaemia in kidney transplant recipients in the first year after transplantation: a cross-sectional study | Inadequate outcomes | Inadequate outcomes | NA |
| Marinhox | 2020 | Transplantation Proceedings | Transplantectomy in the First 3 Months After Renal Transplantation: Experience of a Reference Center | Inadequate outcomes | Inadequate outcomes | NA |
| Naciri Bennani | 2016 | Journal of Nephropathology | Early post-transplant complications following ABO-incompatible kidney transplantation | Population | ABOi transplants only, and inadequate outcomes | NA |
| Niaudet | 2000 | Pediatric Nephrology | Pretransplant blood transfusions with cyclosporine in pediatric renal transplantation | Population | Paediatric cohort, therapeutic pre-transfusions | NA |
| O'Brien | 2012 | Clinical Nephrology | Effect of perioperative blood transfusions on long term graft outcomes in renal transplant patients | Population | 1994-2008 | Transfusions associated with graft loss |
| Prudhomme | 2020 | International Urology and Nephrology | Living-donor kidney transplantation: comparison of sequential and simultaneous surgical organizations | Population | No report of outcomes related to transfusion. 15% ABOi | NA |
| Rogers | 2011 | Transplant International | Desensitization for renal transplantation: Depletion of donor-specific anti-HLA antibodies, preservation of memory antibodies, and clinical risks | Population | HLAi patients only, inadequate outcomes | NA |
| Scornik | 2009 | Transplantation | Effects of blood transfusions given after renal transplantation | Population | Paediatric sub-cohort, also transfusions given >1st year | No association with rejection or DSA |
| Sert | 2013 | Transplantation Proceedings | Anemia in living donor kidney transplantation | Population | 1994-2009, Inadequate outcomes | NA |
| Sharif | 2011 | Experimental & Clinical Transplantation: | Cyclophosphamide exposure pretransplant is associated with complications in the first year after kidney transplant | Inadequate outcomes | Inadequate outcomes | NA |
| Tsujimura | 2018 | Transplantation Proceedings | Effect of Perioperative Blood Transfusions in Renal Transplant Patients | Population | 2009-2016. High proportion of ABOi (26%) | No difference in rejection or function |
| Willis | 2020 | PLoS ONE | Outcomes following kidney transplantation in patients with sickle cell disease: The impact of automated exchange blood transfusion | Population | 1997-2017, inadequate outcomes | NA |

**Table S2. Risk of Bias Assessment using ‘Risk Of Bias In Non-randomized Studies - of Exposure (ROBINS-E)’** Blank boxes, outcome not reported


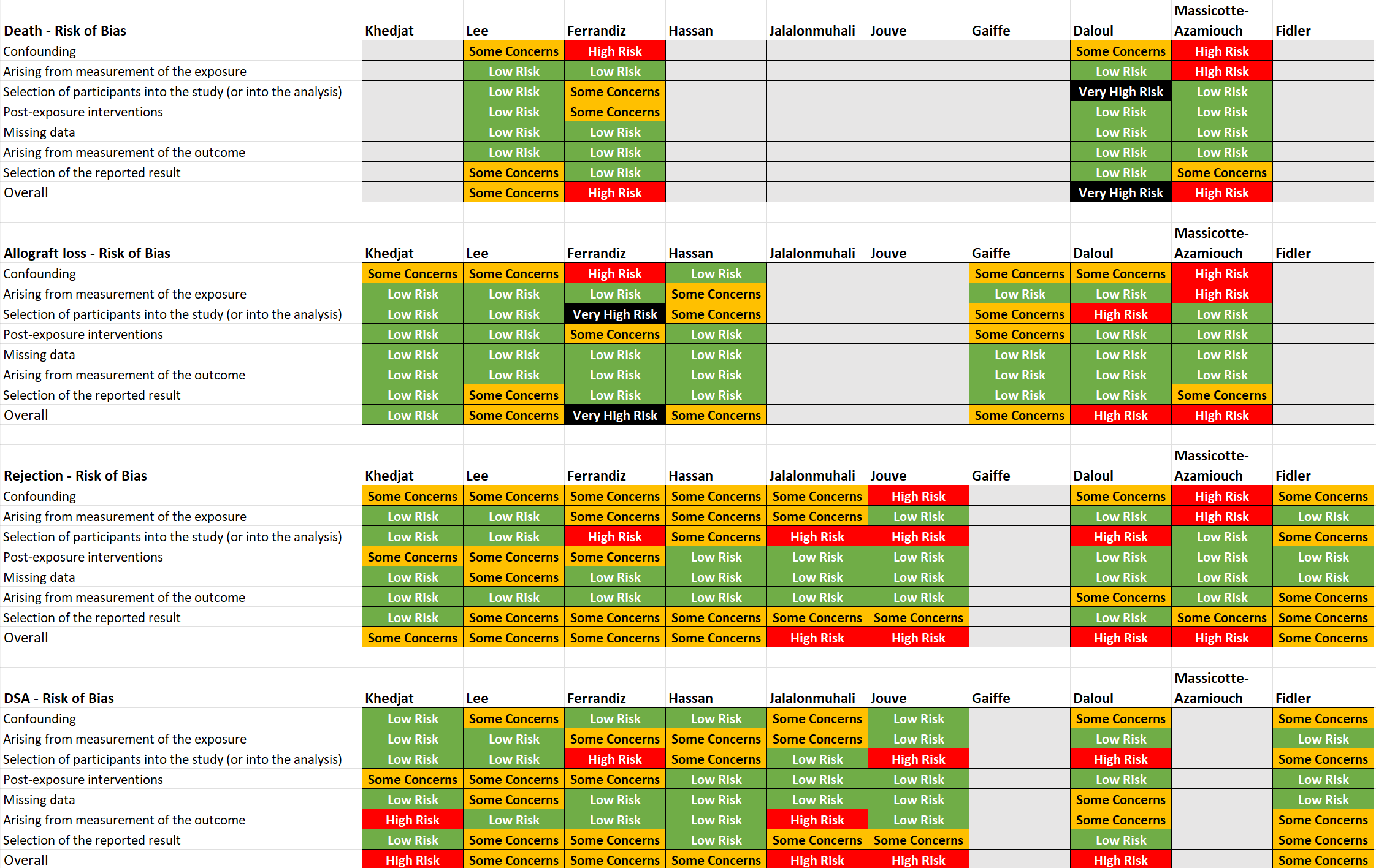

Supplement: Supplementary file 1 [file Table1.docx]
